# The scope for nuclear selection within *Termitomyces* fungi associated with fungus-growing termites is limited

**DOI:** 10.1186/1471-2148-14-121

**Published:** 2014-06-05

**Authors:** Tania Nobre, Bertha Koopmanschap, Johan JP Baars, Anton SM Sonnenberg, Duur K Aanen

**Affiliations:** 1 Laboratory of Genetics, Wageningen University and Research Center, Droevendaalsesteeg 1, Radix West, Building 107, 6708 PB Wageningen, The Netherlands; 2 Plant Research International – Mushrooms, Wageningen University and Research Centre, Droevendaalsesteeg 1, 6708 PB Wageningen, The Netherlands; 3 Currently: ICAAM, University of Évora, Pólo da Mitra Apartado 94, 7002-554 Évora, Portugal

**Keywords:** *Termitomyces*, Ploidy, Polyploid, Levels of selection, Social evolution, Mutualism, Fungi, Mating system

## Abstract

**Background:**

We investigate the scope for selection at the level of nuclei within fungal individuals (mycelia) of the mutualistic *Termitomyces* cultivated by fungus-growing termites. Whereas in most basidiomycete fungi the number and kind of nuclei is strictly regulated to be two per cell, in *Termitomyces* mycelia the number of nuclei per cell is highly variable. We hypothesised that natural selection on these fungi not only occurs between mycelia, but also at the level of nuclei *within* the mycelium. We test this hypothesis using *in vitro* tests with five nuclear haplotypes of a *Termitomyces* species.

**Results:**

First, we studied the transition from a mixture of five homokaryons (mycelia with identical nuclei) each with a different nuclear haplotype to heterokaryons (mycelia with genetically different nuclei). *In vitro* cultivation of this mixture for multiple asexual transfers led to the formation of multiple heterokaryotic mycelia, and a reduction of mycelial diversity over time. All heterokaryotic mycelia contained exactly two types of nucleus. The success of a heterokaryon during *in vitro* cultivation was mainly determined by spore production and to a lesser extent by mycelial growth rate. Second, heterokaryons invariably produced more spores than homokaryons implying that homokaryons will be outcompeted. Third, no homokaryotic ‘escapes’ from a heterokaryon via the formation of homokaryotic spores were found, despite extensive spore genotyping. Fourth, in contrast to most studied basidiomycete fungi, in *Termitomyces sp.* no nuclear migration occurs during mating, limiting the scope for nuclear competition within the mycelium.

**Conclusions:**

Our experiments demonstrate that in this species of *Termitomyces* the scope for selection at the level of the nucleus within an established mycelium is limited. Although ‘mate choice’ of a particular nuclear haplotype is possible during mating, we infer that selection primarily occurs between mycelia with two types of nucleus (heterokaryons).

## Background

Life is organised in a hierarchical fashion. Genes are organised on chromosomes, chromosomes in nuclei, organelles in cells, cells in individuals, and individuals of different species in mutualisms and individuals of the same species in colonies. Entities thus form groups that can become a new unit of selection [1]. However, as long as the constituent entities have some autonomy, lower-level selection can oppose higher-level organisation. For example, selection for fast replication among the cells of multicellular individuals can lead to cancer. Also ‘organelle cancers’ have been discovered that decrease cell fitness and are caused by selection among mitochondrial genomes for fast replication within cells or increased transmission [2,3]. To understand the emergence of higher levels of biological organisation we must understand how the interests of lower-level units became united and the relative importance of selection shifted towards a higher level of biological organisation.

**Figure 1:**
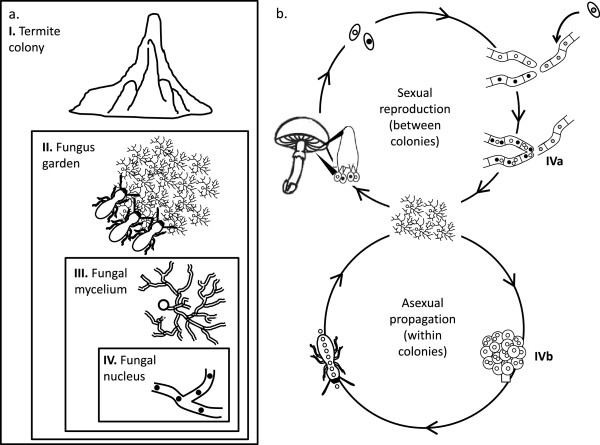
**Schematic representation of the levels of selection acting on the fungal symbionts of a termite colony (A) and the life cycle of species of *Termitomyces *(B). A**. Natural selection may act at the level of colonies (I), fungus gardens within colonies (II), fungal individual (mycelia) within fungus gardens and nuclei within mycelia (IV). **B**. Species of *Termitomyces* have a sexual (upper circle) and an asexual life cycle (lower circle). Sexual reproduction occurs via the production of sexual fruiting bodies (mushrooms), which produce haploid spores. Workers from newly established colonies start a fungus garden from sexual spores. Sexual spores germinate and give rise to mycelia with a single haploid nucleus (homokaryons). Multiple homokaryons can fuse and form a heterokaryon with genetically different nuclei in a mycelium. Within colonies, *Termitomyces* is propagated asexually via the production of asexual spores on nodules, which are inoculated on freshly collected plant material. There are two stages during which selection at the level of the nuclei can occur (indicated as IVa and IVb). First, in the sexual cycle, more than two homokaryons may fuse, so that nuclei are in competition to become part of the heterokaryon that will become dominant during subsequent growth (IVa). Second, during the formation of asexual spores within the colony, a nucleus with a replication or segregation advantage may increase in frequency with time (IVb).

In this article, we study the potential for multi-level selection in the mutualistic fungi cultivated by fungus-growing termites. To what extent does natural selection operate *within* individuals (mycelia) of these fungi? Fungus-growing termites form colonies consisting of thousands of sterile individuals, which cultivate a fungus inside their colony, housed in multiple connected fungus gardens. The termite colonies are founded by a single reproductive pair, and most species rely on horizontal acquisition of fungal symbionts [4-6]. New colonies thus start without a fungus and acquire their fungal symbiont from the environment as basidiospores (sexual spores; Figure 1B). These spores are produced by the sexual fruiting bodies, the mushrooms, which generally are produced seasonally, a few weeks after the termite nuptial flight period [4,7,8]. In contrast to this sexual fungal propagation when new colonies start, fungal propagation within the nest is always asexual, via the continuous propagation of asexual spores (Figure 1B; [9-11]). All colonies screened so far contained a single-strain monoculture of *Termitomyces* [e.g. [9,12].

Natural selection on *Termitomyces* fungi potentially not only acts at the level I. of the colony, but also II. of fungus gardens within colonies, III. of fungal individuals (mycelia) within fungus gardens and IV. of nuclei (and other organelles) within fungal individuals (Figure 1A). We address the potential for selection at the level of nuclei within *Termitomyces* (Figure 1B).

Fungal individuals (mycelia) deviate from other multicellular organisms in three key respects. First, mycelia are not strongly compartmentalised and compartments usually contain multiple haploid nuclei, which can cross the boundaries of compartments [13]. Second, fungal mycelia have a modular organisation and each fragment can reproduce via fission or the formation of asexual spores. Third, fungal mycelia can fuse so that organelles may disperse from one mycelium to another. These characteristics favour selection at the level of the nucleus, and experimental evidence has been found for this hypothesis [14]. *Termitomyces* cells in particular are multinucleate with up to 10 nuclei per cell and thus do not regulate the precise number of nuclei per cell [15]. This is in sharp contrast to most other basidiomycetes, which have special adaptations such as clamp connections to regulate the number and kind of haploid nuclei to be precisely two per cell [16]. We thus hypothesise that *Termitomyces* is prone to nucleus-level selection. In the *Termitomyces* life cycle, selection at the level of the nucleus potentially occurs at two stages. First, in the sexual life cycle, if more than two homokaryons meet, multiple nuclei may compete within the mycelium to become part of the heterokaryon that ultimately becomes the dominant strain of a colony (Figure 1B, IVa). Second, during the asexual stage, nuclei with a replication or a segregation advantage during spore formation can be selected (Figure 1B, IVb). Crucially, nucleus-level selection may favour a nuclear variant even if this variant decreases mycelial fitness, as has been shown in other fungi [17,18]. To test the hypothesis that *Termitomyces* is prone to nucleus-level selection, we performed competition experiments among multiple nuclei, using mixtures of homokaryons and following nuclear segregation in the asexual spores of heterokaryons.

## Results

### Patterns of sexual compatibility between homokaryons

**Figure 2:**
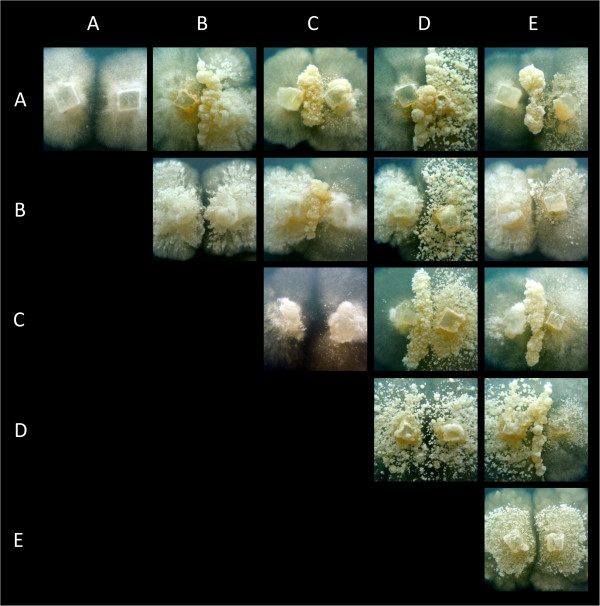
**Interactions between five homokaryotic mycelia of *Termitomyces sp*.** Homokaryon codes **(A to E)** are given along the axes. Distinct zones with increased growth are visible where two different homokaryotic mycelia meet. These zones with increased growth remain local and do not spread across the mycelia. Genetic analysis shows that only the interaction zone has become heterokaryotic, meaning that there is no nuclear migration throughout the homokaryons. Pairings between identical homokaryons are along the diagonal, and do not show these interaction zones.

Using protoplast regeneration, we obtained five homokaryons from three heterokaryons of the *Termitomyces sp.* associated with the termite species *Macrotermes natalensis* (hereafter referred to as *Termitomyces sp.*; Additional file 1: Table S1). These five *Termitomyces* homokaryons were compatible when paired in all 10 pairwise combinations, and led to the formation of a stable heterokaryon. Pairing occurred in a characteristic fashion different from most other basidiomycetes. In all cases, the growth morphology of the contact zone changed, and formed a thick ridge (Figure 2). Genetic analysis of this interaction zone and of both sides of the pairing showed that only the interaction zone had become heterokaryotic, while the rest of the mycelia had remained homokaryotic. Heterokaryons were thus formed upon fusion between the two homokaryons in the contact zone, without the subsequent nuclear migration observed in most other basidiomycetes [16,19].

### Growth patterns of homokaryons and heterokaryons

**Figure 3:**
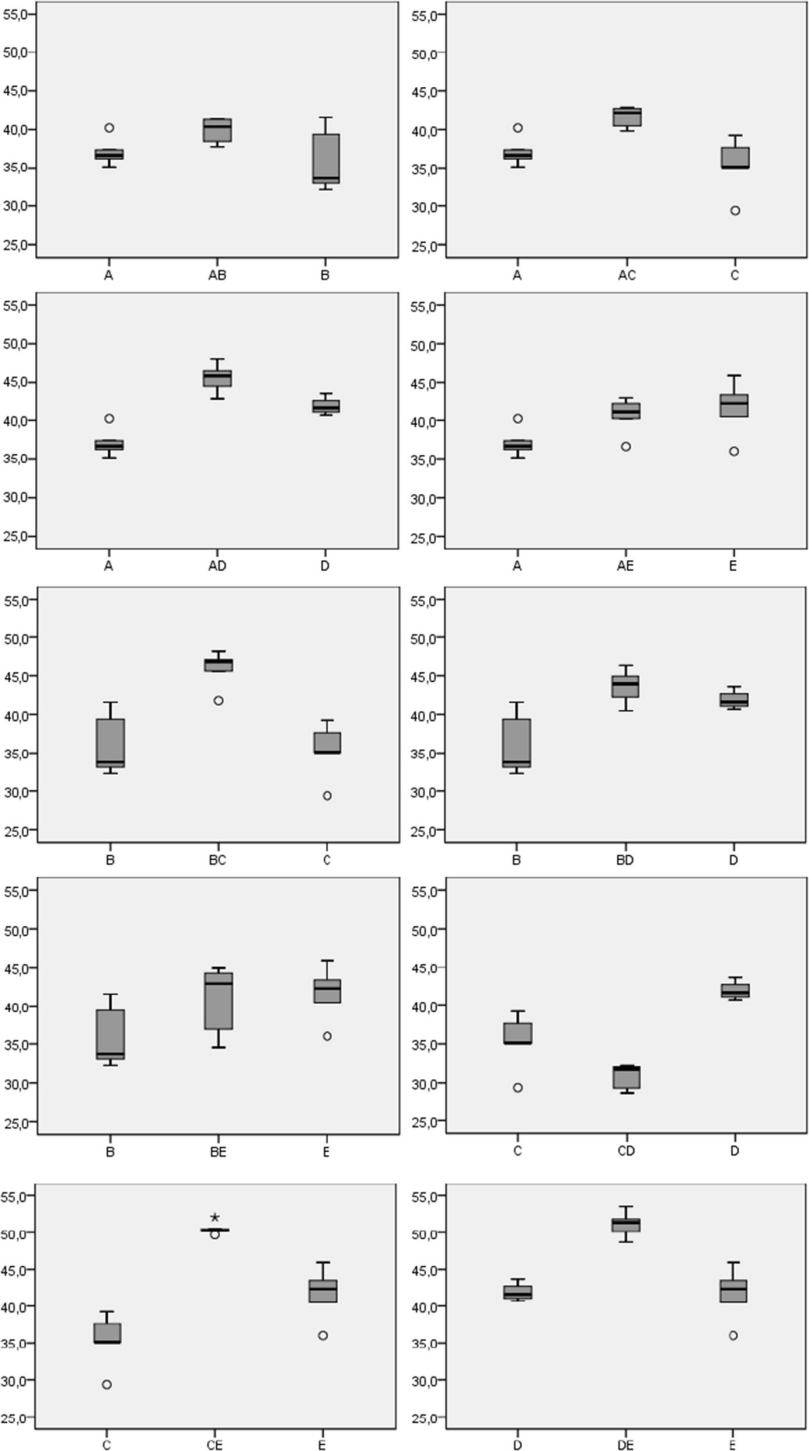
***Termitomyces sp. *mycelial growth.** Boxplot of mycelial growth of the different *Termitomyces sp.* strains after 4 weeks. Each chart represents the heterokaryon (in the middle) surrounded by its two component homokaryons. ○ outliers (≤3x interquartile range [IQR]); **★** extremes (≥3x IQR).

Overall, heterokaryons had a significantly higher growth rate than homokaryons (Figure 3, GLM ANOVA, with karyotic state as main effect, nuclear haplotype as cofactor; Z = 14.29 with *p* = 0.00, Additional file 1: Table S3). For 9 of the 10 combinations of two nuclei, the mycelial growth rate of the heterokaryon was higher than the average of its two component homokaryons. The only exception was heterokaryon CD, which had a lower growth rate than both its constituent homokaryons C and D. A more conservative, non-parametric test, assuming an equal probability of a higher or smaller growth rate of the heterokaryon than that of the mean growth rate of its two component homokaryons, already gave a significant result [*p *(9 times higher and one times smaller) = 10 X (0.5)^10^ = 0.0098].

**Figure 4:**
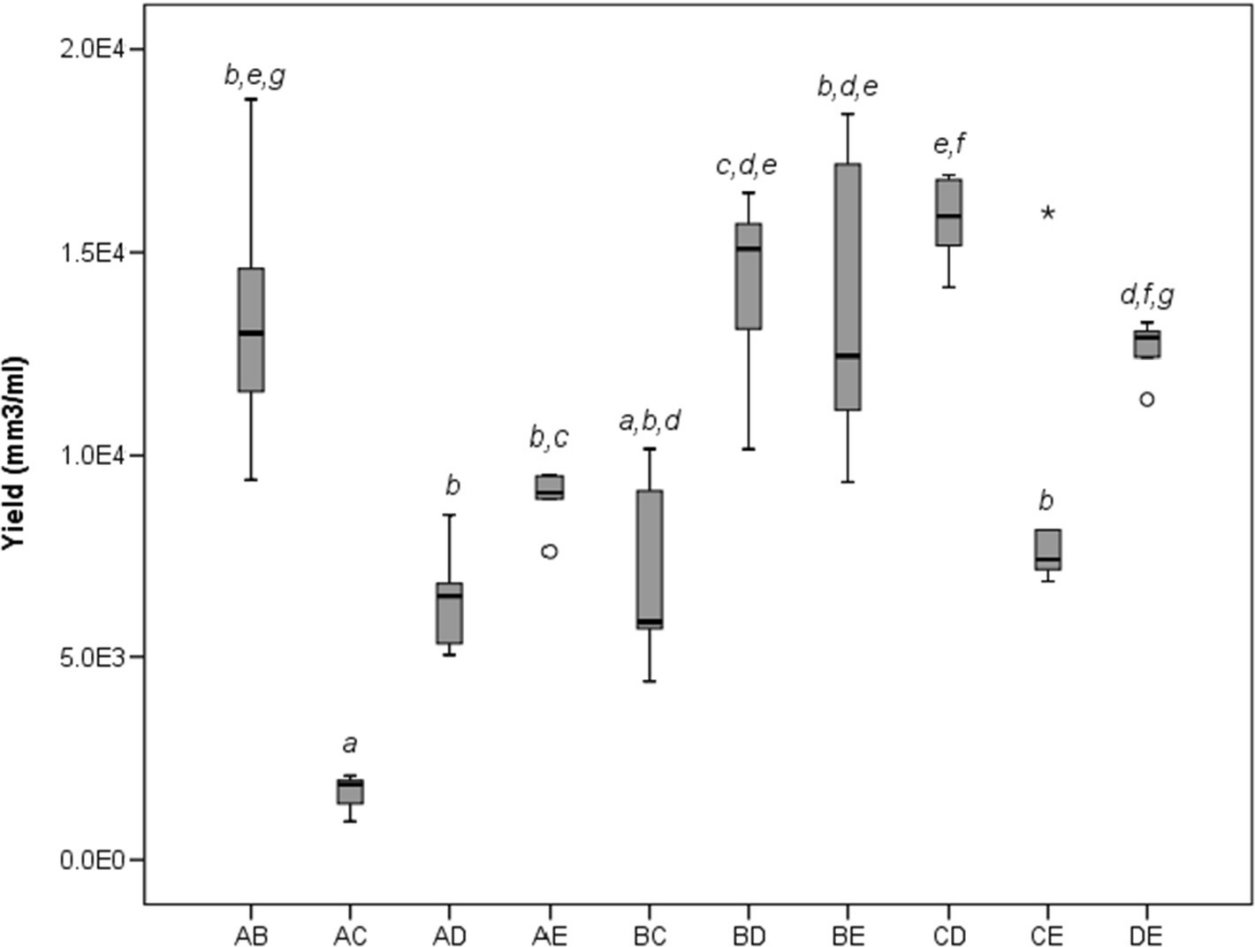
**Nodule formation in the 10 heterokaryons.** Boxplot of yield (mm^3^ nodules/ml) of the 10 heterokaryons (n = 5); ○ outliers (≤3x IQR); **★** extremes (≥3x IQR); averages followed by the same letters are not significantly different (p > 0.05).

The production of asexual spores by homokaryons was always lower than that by heterokaryons. However, especially for homokaryons, nodule formation was highly unstable, ranging from absent to a few nodules for the same strain under the exact same growing conditions, so we refrained from quantification. The generated heterokaryons showed significant differences in nodule formation although generally the variance between replicates within strains was high (Figure 4; Kruskal-Wallis test [variances not homogeneous] *p* < 0.001).

### Patterns of heterokaryon formation and subsequent selection

The initial stages of a fungus comb originating from sexual spores collected from the environment were simulated under laboratory conditions by mixing the homokaryons both in equal and in unbalanced proportions (with one strain in the majority). After one week, the asexual spores formed were harvested and 10% was inoculated on new plates (see Methods for details).

**Figure 5:**
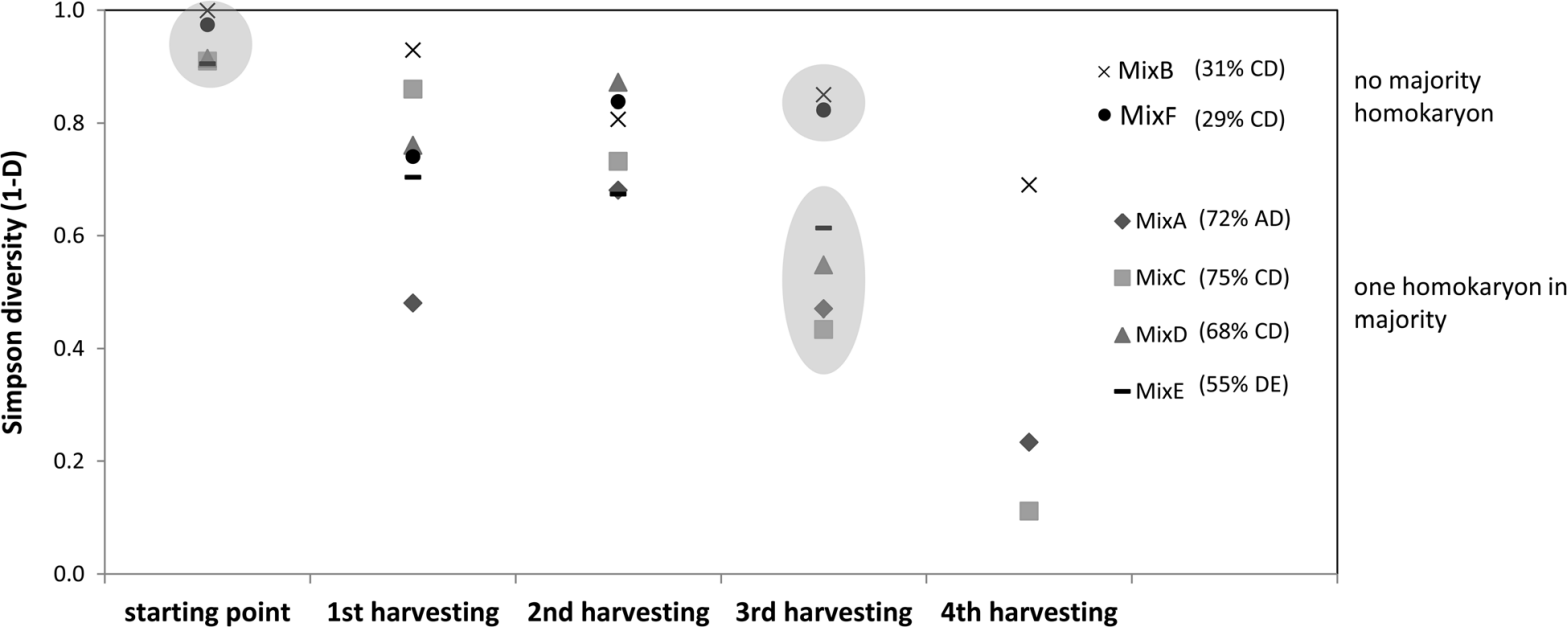
**Simpson diversity index (1-D) at the start of the experiments and throughout the experiment.** The most common heterokaryon at the third harvesting is indicated, preceded by its percentage at this last stage. At the third harvesting, the two treatments with different starting frequencies were significantly different in diversity (grey shape encompasses non-significantly different diversity measures assessed by a pairwise Student’s t test). Due to contamination, for some treatments, no data were obtained for the fourth harvesting.

In most cases, the homokaryons were rapidly heterokaryotized. However, importantly, we never found a heterokaryon with more than two nuclei. Already at the first harvesting, the proportion of remaining homokaryons usually was small, although some replicates retained up to 30% homokaryons. Surprisingly, on some plates, a small proportion of homokaryons remained until the third harvest (Additional file 1: Figure S4 and S5). As expected, over the course of multiple harvestings, diversity significantly decreased (Figure 5) leading to the dominance of a single heterokaryon in the mixture. Furthermore, at the end of the experiment, a significantly different diversity was observed between the two treatments with different starting frequencies. If one homokaryon was in the majority, diversity decreased faster than if the initial mixture consisted of equal frequencies of homokaryons. This shows that although the ultimate result will likely be a single heterokaryon, the speed of this transition depends on the initial situation. The dominant heterokaryon always consisted of the nucleus from the majority homokaryon, associated with a specific nucleus [D]. When this nucleus was in the majority homokaryon itself (MixD), the resulting heterokaryon was the same heterokaryon that became dominant in the mixes with no single most frequent homokaryon [CD].

### Nuclear composition of asexual spores

The number of nuclei per asexual spore was determined for the two heterokaryons AD and DE by comparing the fluorescence image showing the nuclei and the normal bright field image showing the cell walls and septa between adjacent cells. The average number of nuclei was similar in the asexual spores of both tested heterokaryons, with a higher deviation from evenness in the spores originating from heterokaryon DE (Table 1). Obviously, an odd number of nuclei in a spore implies a deviation from a 50% ratio of both nuclei in that spore.

**Table 1:** Nuclear distribution in asexual spores of two heterokaryons

	**AD**	**DE**	**Even # of nuclei**	**Odd # of nuclei**
Mode	4	3		
Average	3.7	3.6		
Max	6	6		
Min	2	2		
n	106	105		
n (even)	56	37		

The image on the left shows two nuclei in a spore and the one on the right five nuclei in a spore, as revealed by nuclear staining with DAPI. This implies that the nuclear distribution in the asexual spores can deviate from 50%. Note that the proportion of even nuclei per spore varies greatly between the two tested heterokaryons.

Considering a mode of three nuclei per asexual spore, the nuclear identity of 12 single spore cultures is sufficient to show, with a probability of 95%, that the absence of homokaryons in the sample is higher than expected if nuclear segregation occurs randomly (see Additional file 1 for details). Likewise, for a mode of four, a sample of 24 single spore cultures is needed. We genotyped 100 single spore cultures per generated heterokaryon and no single homokaryon was found, meaning that all asexual spores analysed contained the two nuclear types.

## Discussion

Natural selection simultaneously acts at different levels in the hierarchy of life. To explain higher levels of biological organisation, we need to understand how the relative importance of lower-level selection decreased. A colony of fungus-growing termites is a nice illustration of the hierarchical nature of life (Figure 1). Earlier research had shown that an adult termite colony is associated with a single clone of *Termitomyces*[9]. However, the extent to which within-mycelium selection among multiple nuclei could occur was still unknown. Here we clearly show that the scope for selection at the level of the nucleus is restricted in the *Termitomyces* species associated with the termite *Macrotermes natalensis*. Only during mating, a homokaryon may have a preference for a particular nucleus, which can be considered as an example of ‘female choice’ [20,21]. Below we outline in more detail the consequences of the reported results for the evolution of this mutualism and for multi-level selection in general.

### In contrast to most basidiomycete fungi, *Termitomyces* has no nuclear migration

Pairwise interactions between the five different homokaryons of *Termitomyces sp.* showed that these were sexually intercompatible in all combinations, confirming that the *Termitomyces* strains associated with the termite species *Macrotermes natalensis* all belong to a single biological species [12,15,22]. The high degree of sexual intercompatibility implies a high diversity of mating-type alleles, similar to most basidiomycetes studied so far [16,23]. In contrast to most other basidiomycetes, however, there is no nuclear migration following fusion among the tested *Termitomyces sp.* homokaryons. It is tempting to ascribe this difference with non-cultivated heterothallic fungi to their symbiotic lifestyle. The way the fungus is cultivated by inoculating asexual spores in a high density [10,24] implies that opportunities for fast radial growth and extensive nuclear migration in an established colony are limited. Instead, there is a premium on fast reproduction, as this increases the representation in the next inoculum. Assuming a trade-off between nuclear migration and fast reproduction, selection for fast reproduction implies selection against nuclear migration or, alternatively, the absence of selection for nuclear migration may have eroded the genes required for nuclear migration. Instead of nuclei migrating themselves throughout the mycelium, termites thus accomplish nuclear migration by transporting the nuclei via the asexual spores throughout the fungus comb, which then may grow out and produce new spores, or additionally donate one of their nuclei to unfertilised mycelia via so-called Buller pairings [25]. A different species, *Agaricus bisporus*, does not show nuclear migration either [26]. However, this species is secondarily homothallic, so that spores normally contain two compatible nuclei, and the frequency of outcrossing is low [27]. Therefore, and similar to *Termitomyces sp.*, there is no strong selection on nuclear migration in that species.

### Mycelia consist of two, and never more than two, nuclei

Most basidiomycete fungi have mechanisms to regulate the distribution of the nuclei in a heterokaryotic mycelium, leading to a so-called dikaryon, with precisely two genetically different nuclei per cell [16]. Since *Termitomyces* species do not regulate the precise number of nuclei per cell [15], we inferred that mycelia of *Termitomyces* might consist of more than two genetically different nuclei. This has been observed in another species with multinucleate cells, *Heterobasidion annosum*, where mycelia with three nuclear types have been isolated [28]. In contrast to the latter observations, however, all heterokaryons examined in this study always consisted of two genetically different nuclei, and never more than two. This is consistent with the evidence obtained so far based on sequence data of natural heterokaryons of *Termitomyces*[12,15,22,29]. Therefore, despite the absence of regulation of the number of nuclei per cell, the identity of nuclei is strictly regulated, and the stable coexistence of more than two nuclei in a single mycelium is precluded.

### Heterokaryons produce more spores and have a higher growth rate than homokaryons

Although it is often assumed that heterokaryons have a higher competitive fitness than homokaryons, empirical data give mixed results. For example, whereas Simchen and Jinks [30] found higher growth rates for heterokaryons of *Schizophyllum commune*, Clark and Anderson [31] found the opposite. Hiscox and co-workers [32] found comparable competitive abilities among homokaryons and heterokaryons in the species *Trametes versicolor*. In this study, overall, heterokaryons had a significantly higher growth rate than homokaryons, although the differences were small. Furthermore, heterokaryons consistently produced more asexual spores than homokaryons (Figure 3). This implies that even if a nucleus would ‘escape’ from the heterokaryon to form a homokaryon, this homokaryon would be out competed by heterokaryons over time. We have earlier shown that positive frequency-dependent selection occurs among heterokaryons [9]. We now show that -under the conditions of our experiment- spore production and to a lesser extent growth rate are determining factors for the success of a heterokaryon starting with equal frequencies:

1) The most successful heterokaryon of all ten heterokaryons (CD), winning almost all the competitions, produced most spores, but had the lowest growth rate. This suggests that spore production may be more important than mycelial growth rate during the heterokaryotic stage;

2) The next most successful heterokaryons -DE and AD- both had a high growth rate and moderate to high spore production.Furthermore, our experiments show that being in the majority as a homokaryon, guaranteed the presence of that nucleus in the most dominant heterokaryon at the end of the experiment. Strikingly, nucleus D was part of all the most successful heterokaryons. Homokaryon D was the only homokaryon that consistently produced spores (Figure 2). This suggests that spore production during the homokaryotic stage may also be of relevance.

Almost certainly, growth rate and spore production will differ on natural substrates and, therefore, the ranking of heterokaryons as well. For example, nodules not only are a source of new inoculum and food, but also of fungal enzymes, which are mixed with the substrate [33,34]. This means that other heterokaryons may have a selective advantage on different substrates and under different conditions than the strains here. However, our experiments demonstrate the consequences of selection on the basis of asexual spore production, and that there is limited scope for nuclear competition within mycelia.

### Nuclei cannot cheat by monopolising the spores

Despite extensive sampling effort, we have not found any homokaryotic spore from heterokaryotic cultures. We therefore conclude that no nuclear segregation occurs among asexual spores. In other fungi with a less strict control of the number of nuclei per cell, such cases have been documented [21,35-37]. So, although the number of nuclei per cell [15] and per asexual spore are not strictly regulated and the frequencies of both types often differ, the asexual spores of a heterokaryotic culture always receive both nuclear haplotypes. Nuclear distribution among spores thus appears to be regulated by some unknown mechanism.

### The homokaryon stage is short-lived in nature

Our results demonstrate that, starting with a mixture of homokaryons, heterokaryons with two nuclei arise quickly. Subsequently, drift and positive frequency dependent selection are likely to lead to a single heterokaryon, and all colonies studied so far indeed had a single heterokaryon [9,24]. The symbiont units among which competition occurs thus primarily are the heterokaryons, and not the homokaryons nor the nuclei within mycelia. The scope for selection between homokaryons and between nuclei during the start of a colony, when sexual interactions occur between multiple homokaryons, is therefore limited*.* Only during mating, a homokaryon may have a preference for a particular nucleus, which can be considered as an example of ‘female choice’ [20,21].

In the light of these results, it is striking that sometimes homokaryons persisted up to the 3rd harvesting. However, in pairwise interactions between homokaryons, we noticed that successful mating became less frequent in later stages of the experiment (results not shown). Although we do not have an explanation for this finding, this reduction in successful mating could explain the long-term persistence of homokaryons in our experiments.

### Increases in ploidy are a common theme in mutualistic symbionts

It remains to be demonstrated to which extent the presence of multinucleate cells and spores, which are different than their non-mutualistic sister groups [38], are functionally related to the mutualistic lifestyle of this fungus. However, it is remarkable that completely unrelated symbionts in other mutualisms show convergent increases in ploidy. For example, the unrelated fungi cultivated by leaf-cutting ants, also lack mechanisms to restrict the number of nuclei per cell to two, and have multinucleate cells (and apparently sometimes more than two genetically different nuclei per cell; [39]). Furthermore, in other examples of mutualistic symbiosis, such as between plants and arbuscular mycorrhizas, the ploidy of the fungal symbionts also has dramatically increased with many nuclei per cell and per asexual spore (although the extent to which these nuclei are genetically different is a matter of debate [40-42]). This pattern of increased ploidy in symbionts can even be seen in the two main classes of endosymbionts in eukaryotic cells, plastids and mitochondria, which have highly increased ploidy levels with around 10 genomes per endosymbiont [43]. Even our own crops fit into this general picture, as most of them are polyploids [44]. This striking similarity between completely independent examples of mutualistic symbiosis suggests that increased ploidy of symbionts has been selected as a consequence of a symbiotic lifestyle, possibly because of increased demands from the host on productivity of the symbionts and relaxation of the selective constraints found in non-mutualistic relatives. Despite the high number of nuclei per cell and per spore, however, our study demonstrates that there are only two kinds of haploid nuclei per individual.

## Conclusions

We demonstrate that the scope for selection at the level of the nucleus for the mutualistic fungi cultivated by fungus-growing termites is restricted, despite a less strict regulation of the number of nuclei per cell and per asexual spore: 1. heterokaryons produced more asexual spores than homokaryons, showing that any homokaryotic escape from a heterokaryotic culture would be outcompeted; 2. nuclei cannot monopolise the asexual spores as asexual spores from heterokaryons always had both nuclei; 3. in contrast to most other basidiomycete fungi, no nuclear migration occurred upon mating between homokaryotic mycelia; 4. despite extensive efforts, i.e. by maximising contact between multiple compatible homokaryons in a high density, the resulting heterokaryons always had two and never more than two nuclei.

## Methods

### Homokaryon regeneration

As we did not have access to fresh fruiting bodies of *Macrotermes natalensis*, we could not obtain homokaryons from sexual spores. Therefore, we regenerated homokaryons from heterokaryotic strains using protoplast regeneration [45]. Using previously described methods [22], we first isolated heterokaryotic cultures of *Termitomyces* from three colonies of the species *M. natalensis* collected in South Africa in 2008 (Additional file 1: Figure S1). This species is associated with a unique *Termitomyces* lineage, and genetic data show that strains of this lineage belong to a single biological species [9,12,15,22]. The cultures were isolated on standard malt yeast extract agar (MYA; per liter demi water: 20 g malt extract, 2 g yeast extract, 20 g agar) [22] and stored in -80C in a mixture of pepton-glycerol (30% glycerol, 0.7% neopepton). Upon recovery of the heterokaryons from the freezer, homokaryons were generated from these strains using protoplast regeneration [45]. As this method relies on mycelial outgrowth, a nucleus with a recessive deleterious mutation will be underrepresented in the recovered homokaryotic mycelia. For three heterokaryons, we succeeded to recover five of the six possible homokaryons (Additional file 1: Table S1).

To test the karyotic state of the protoclones, DNA was extracted from mycelium using a CTAB DNA isolation method. The highly variable intron in Elongation Factor 1 alpha (EF1-α) and part of the nuclear ribosomal region including the first internal transcribed spacer (ITS1), the 5.8S RNA gene and the second internal transcribed spacer (ITS2) were amplified using standard PCR reactions (primers and procedure as in [29]). Direct sequencing of the PCR product was performed by MWG Biotech. By comparison with the electropherograms of the heterokaryotic parental strain, we confirmed the recovery of one or both homokaryotic strains. The regenerated homokaryons were used in the following four analyses/experiments.

### Patterns of sexual compatibility

To test sexual compatibility and patterns of heterokaryon formation, we made all 10 pairwise combinations of the five recovered homokaryons by placing inocula side-by-side, with ca. 50 mm of space in between, on 5X diluted MYA medium. After four weeks of growth, the pairings were inspected and photographed. Samples of the contact zone and the sides of pairings were subcultured and genotyped, to test their nuclear status.

### Growth measurements and nodule formation

To test mycelial growth differences among homokaryotic and heterokaryotic strains, cultures of the regenerated *Termitomyces sp.* homokaryons and of newly created heterokaryons were grown on a 5X diluted MYA medium (5 replicates per strain) and incubated at 25°C. Mycelial growth was measured after four weeks. Plate surfaces were photographed with the Molecular Imager® Gel Doc XR System from Bio-Rad, using fixed conditions of aperture size and shutter speed. The surface area covered with mycelia was estimated using the software ImageJ (http://rsb.info.nih.gov/ij) and linearized (square root transformation).

To measure asexual spore production of all strains, approximately 20 mm^3^ of the nodule biomass was scraped off from a two-week old culture, suspended in 500 μl saline with a small mortar fitting in the bottom of an Eppendorf tube. Per plate, 50 μl of spore suspension was inoculated and distributed using five sterile glass beads. After 10 days of growth, plate surfaces were photographed as above and the surface area covered with nodules was estimated following the protocol described in [9].

The variables analysed (mycelial growth, nodulation and nuclear distribution in asexual spores) were summarized using boxplots or by describing their distribution. Nodule formation was described as asexual spore yield, which was calculated as volume $(Y={\left(\sqrt{A}\right)}^{\wedge }3/\sqrt{n)}$ based on the area covered with nodules (*A)* and number of nodules (*n*) as estimated by the ImageJ software on size calibrated images. Differences in mycelial growth were analysed through ANOVA by a general linear model (GLM), using mycelial linear growth as dependent variable and the karyotic state as fixed factor with two states heterokaryotic or homokaryotic. The nuclear components of the homokaryons and heterokaryons in the analysis were coded and entered into the analysis as covariates (in this analysis, considered as other independent variables of interest).

### Homokaryon mixing experiment

Stock suspensions of asexual spores of the five homokaryons were made as described above. Six spore suspensions were made: a mixture of the five homokaryon strains in equal proportions and the five possible mixtures where one homokaryon constituted 50% of the inoculum volume and the remaining four 12.5% each. The rationale for the latter treatment was that the 50% strain would most often function in a receiving role, so that the winning partner nucleus could reflect ‘female choice’ in this treatment [21]. This would correspond to a situation, where a single spore arrived first, giving a head start to a single homokaryon with that nucleus. Because spores are collected from the environment, it is likely that multiple spores are brought into the nest: fruiting occurs a few weeks after the termite nuptial flight period [4,7,8], meaning some synchronization in fruiting and in spore availability.

The numbers of colony-forming units (CFU) per suspension were quite similar between strains with the exception of strain B (4 times fewer CFU’s; Additional file 1: Table S4). As a consequence, the targeted combinations with B in majority correspond to the mixture of five homokaryon strains in equal proportions. The other combinations with unequal shares resulted in one of the strains constituting ~57% of the inoculum volume, three strains 13% each and the remaining (strain B) with 4%.

After 10 days incubation, the nodule biomass of half of the plate was scraped off ensuring capturing plate diversity, suspended in 500 μl saline and mixed as described above. Plates were inoculated in the same way as described above until four harvestings were completed, simulating asexual spore propagation in a termite society. Because we wanted to identify and characterize asexual spores diversity within the mixtures, at each harvesting point, plates with different spore concentrations (5, 25, 125 times diluted) were inoculated to allow isolation of individual nodules. After one week of growth, 30 nodules per initial mixture were isolated on MYA plates (Additional file 1: Figure S2), allowed to grow for 10 days and the DNA of these pure cultures was extracted as described above. Using the KASP SNP genotyping system (KBiosciences) with specific primers designed according to the product instructions, the isolated nodules were genotyped at known single nucleotide polymorphisms (SNPs) previously selected from the homokaryotic sequences obtained. Genotyping at three SNPs (2 from EF1α and 1 from ITS1) was sufficient to distinguish all 10 possible heterokaryons (Additional file 1: Table S2).

As a measure of diversity of all genotypes in the mixes (thus including homokaryons and heterokaryons) at the different harvesting points, we used the Simpson's Diversity Index (1-D) and tested for pairwise significant differences using Student’s *t*-test (α = 0.05). The starting diversity of heterokaryons was estimated assuming random pairwise association between the homokaryons in a mixture.

### Asexual spores and nuclei

Homokaryotic isolates observed in the later stages of the mixing experiment could either result from non-fused homokaryons or re-emergence of homokaryons from heterokaryons via homokaryotic asexual spores. For two of the three heterokaryons that reached the majority in the end of the mix experiments (the third strain was lost) we tested if heterokaryons produced any homokaryotic spores. For that, we determined 1) the number of nuclei per asexual spore and 2) the nuclear genotype of the asexual spores. For 1), asexual spores were scrapped off and immediately placed in 10 μl of the florescent dye DAPI, and we used fluorescence microscopy to visualise the nuclei ([46,47]; details in Additional file). For 2), 100 single spore cultures were genotyped. The material to be genotyped was prepared as follows: stock suspensions of asexual spores were made as described for heterokaryotic yield, suspensions were filtered through a glass wool funnel and washed four times, each time with 500 μl saline, to remove any mycelial fragments or clumps of spores. Two further five times dilutions of spore suspension were made and 50 μl was inoculated per Petri dish and distributed using five sterile glass beads. After one week of growth the nodules were isolated and genotyped for nuclear identification, as described above.

Our null hypothesis was that nuclei segregate randomly among the asexual spores and the alternative hypothesis that nuclei are distributed in a regulated fashion to maintain two different nuclei per spore. For all statistical tests, the significance level considered was α = 0.05.

## Availability of supporting data

The data set supporting the results of this article is available in the Dryad repository, Dryad doi:10.5061/dryad.b28k2 [48] [http://datadryad.org/resource/doi:10.5061/dryad.b28k2].

## Competing interests

The authors declare no competing interests.

## Authors’ contributions

TN and DKA conceived and designed the study, collected the samples, and wrote the manuscript. TN carried out all the experiments. BK participated in the genotyping essays. JJPB and ASMS organized the protoplasting. DKA coordinated the study. All authors read and approved the final manuscript.

## Supplementary Material

Additional file 1 **Supplemental material.** Details on the methodology and results. **Figure S1.** Sampling: two colonies from one locality in Pretoria (S25 43 45.2 E28 14 05.8 and S25 43 45.9 E28 14 08.9) and one from Mookgophong, which is 158 kilometers north of Pretoria (S24 40 30.5 E28 47 50.4). Maps from GoogleMaps.com. **Figure S2.** Sampling procedure in the mixing experiments. **Table S1.** Collected heterokaryons and its correspondence with the recovered homokaryons. **Table S2.** Polymorphisms that allow the discrimination of the 10 different *Termitomyces* heterokaryons used in the experiments. SNP numerical code corresponds to the position of the polymorphism using the GenBank DQ437019 as EF1-α reference and AB073531 as ITS reference. **Table S3.** Statistic values for the univariate analysis of mycelial growth, GLM ANOVA with karyotic state as main effect, nuclear haplotype as cofactor. **Figure S3.** Representative images of nodule formation in the 10 heterokaryons. **Table S4.** CFU’s present in the stock suspensions as determined on counting plates and the effective proportions of the different homokaryons in the different combinations. **Figure S4.** Representation of the different heterokaryons present at each harvesting. **Figure S5.** Representation of the different heterokaryons present at each harvesting. **Table S5.** Simpson diversity index (1-D) per mixture, discriminated following harvesting and replicate. Differences between replicates are significant at p<0.05, following a Student’s t-test. Click here for file
