# Supplementary material for: The scope for nuclear selection within Termitomyces fungi associated with fungus-growing termites is limited
Source: BMC Evol Biol. 2014 Jun 5;14:121. doi: 10.1186/1471-2148-14-121 (PMC4085734; doi:10.1186/1471-2148-14-121)
Supplement: Additional file 1 — Supplemental material. Details on the methodology and results. Figure S1. Sampling: two colonies from one locality in Pretoria (S25 43 45.2 E28 14 05.8 and S25 43 45.9 E28 14 08.9) and one from Mookgophong, which is 158 kilometers north of Pretoria (S24 40 30.5 E28 47 50.4). Maps from GoogleMaps.com. Figure S2. Sampling procedure in the mixing experiments. Table S1. Collected heterokaryons and its correspondence with the recovered homokaryons. Table S2. Polymorphisms that allow the discrimination of the 10 different Termitomyces heterokaryons used in the experiments. SNP numerical code corresponds to the position of the polymorphism using the GenBank DQ437019 as EF1-α reference and AB073531 as ITS reference. Table S3. Statistic values for the univariate analysis of mycelial growth, GLM ANOVA with karyotic state as main effect, nuclear haplotype as cofactor. Figure S3. Representative images of nodule formation in the 10 heterokaryons. Table S4. CFU’s present in the stock suspensions as determined on counting plates and the effective proportions of the different homokaryons in the different combinations. Figure S4. Representation of the different heterokaryons present at each harvesting. Figure S5. Representation of the different heterokaryons present at each harvesting. Table S5. Simpson diversity index (1-D) per mixture, discriminated following harvesting and replicate. Differences between replicates are significant at p<0.05, following a Student’s t-test. [file 1471-2148-14-121-S1.docx]

**Supplemental material**

**The scope for nuclear selection within *Termitomyces* fungi associated with fungus-growing termites is limited**

Nobre T, B Koopmanschap, JJP Baars, ASM Sonnenberg and DK Aanen

**Methods**


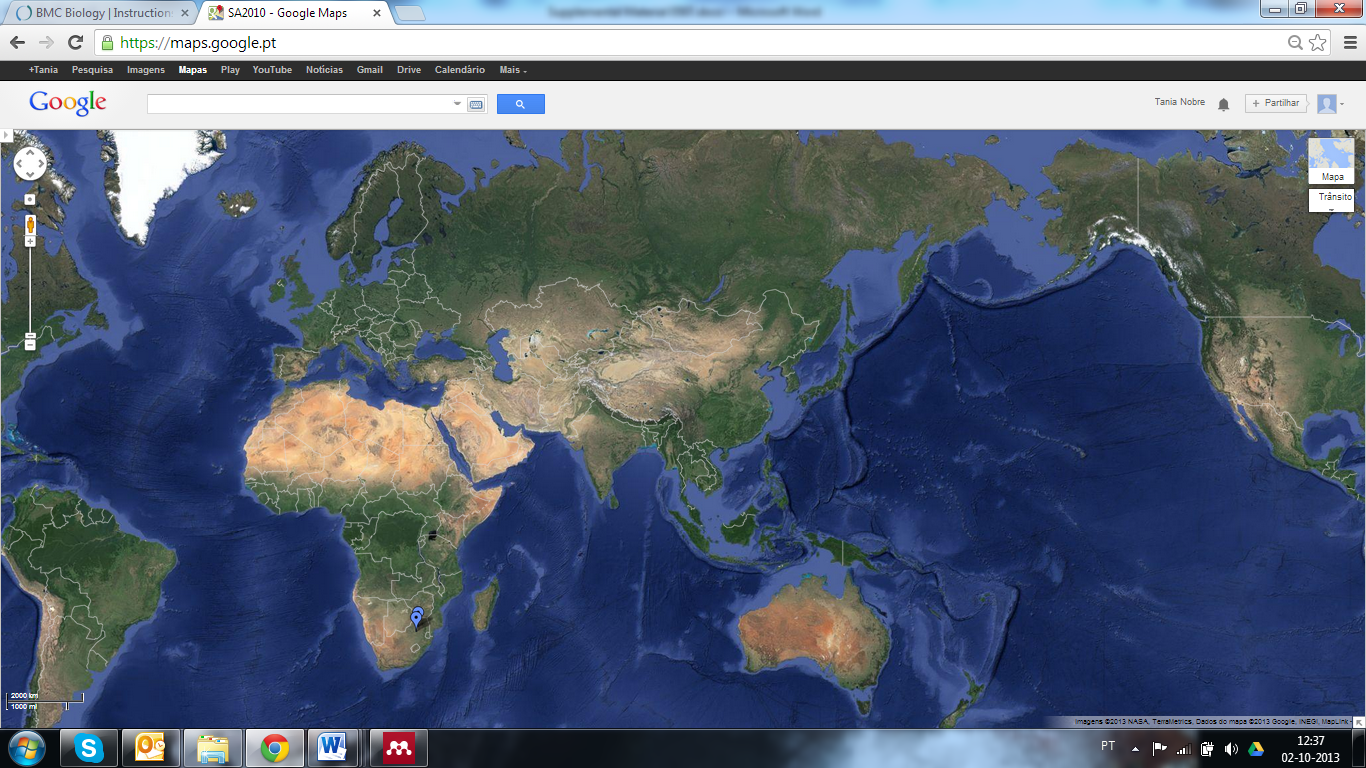

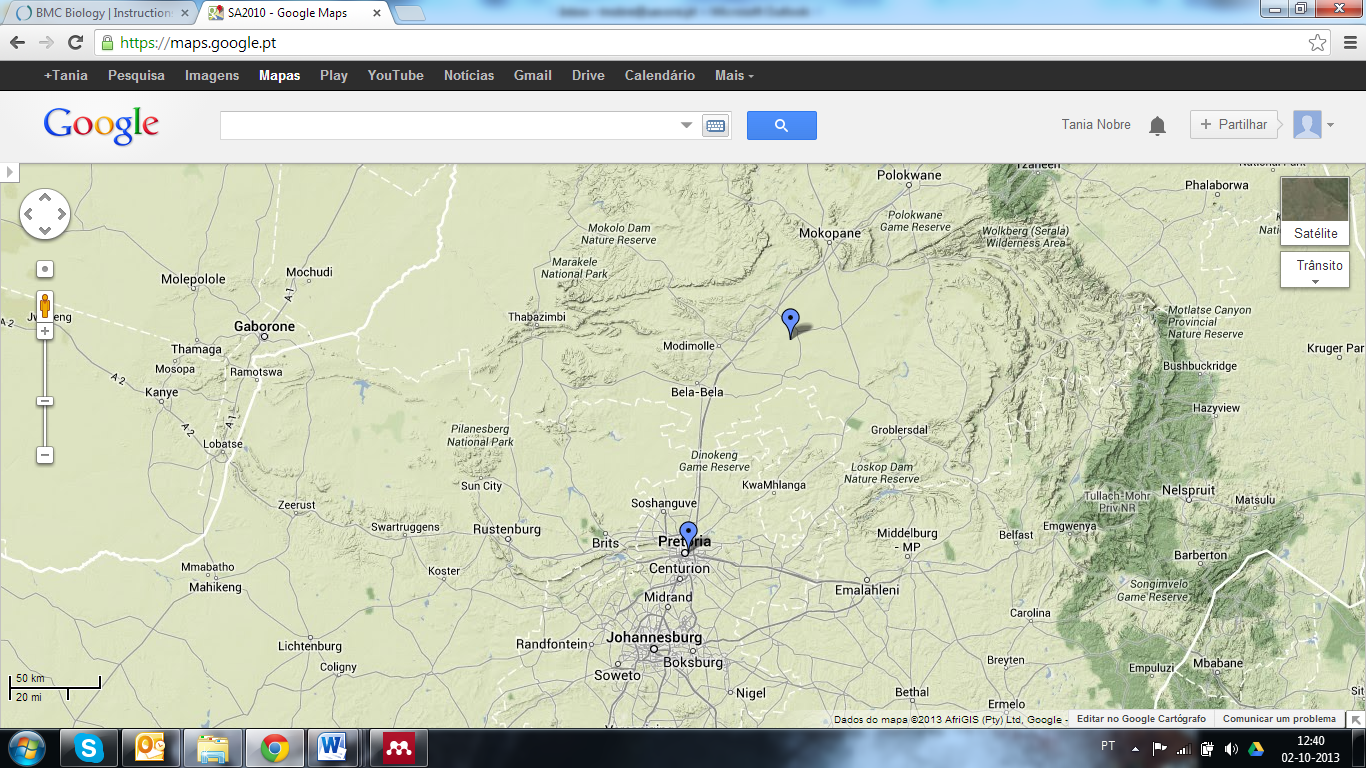


***ca.* 158 km**

**Figure S1:** Sampling: two colonies from one locality in Pretoria (S25 43 45.2 E28 14 05.8 and S25 43 45.9 E28 14 08.9) and one from Mookgophong, which is 158 kilometers north of Pretoria (S24 40 30.5 E28 47 50.4). Maps from GoogleMaps.com

1^st^ harvesting

2^nd^ harvesting

3^rd^ harvesting

4^th^ harvesting


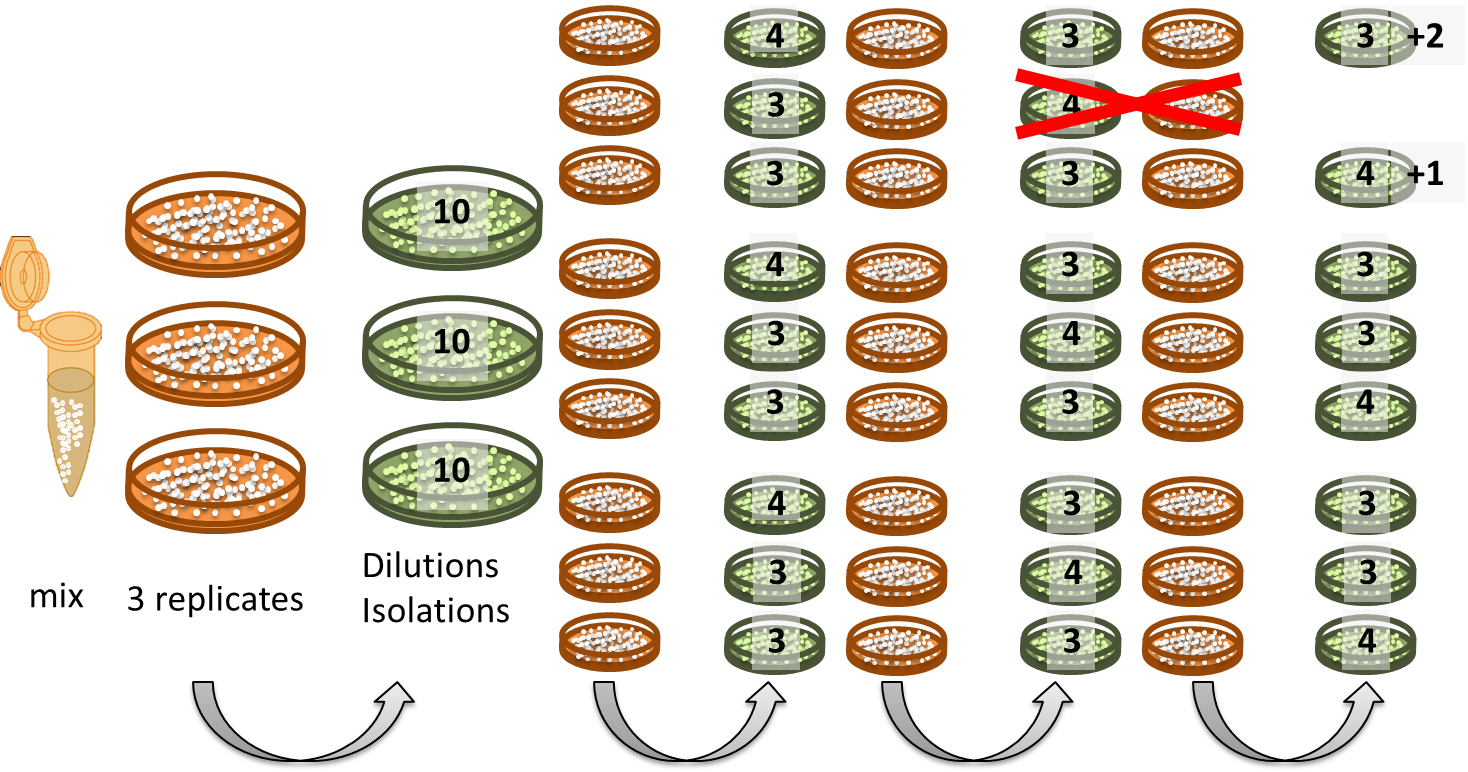


**Figure S2:** Sampling procedure in the mixing experiments. After the 10 days incubation period at 25 °C the nodule biomass of half of the plate was scraped off, suspended in 500 μl saline and mixed by vortexing. 50μl was applied per (90 mm diam.) Petri dish with diluted MYA, and spread over the plate with five sterile glass beads. For the 1^st^ harvesting, per mixture replicate, three further replicates were inoculated. This process was maintained (with no further replication) until 4 harvestings were completed, simulating asexual spore propagation in a termite society. Each time, plates with different spore concentrations (5, 25, 125 diluted) were inoculated for nodule isolation after a week of growth (30 nodules per mixture combination were isolated into MYA). This setup, is likely to promote diversity compared to what occurs in a fungus-growing termite society (thus resulting in increased time to stabilization). However, this setup has the advantage that the experiment can be continued even if a certain line is lost due to contamination or other reasons.

**Table S1**: Collected heterokaryons and its correspondence with the recovered homokaryons

| Collected heterokaryon | Recovered homokaryon | |
| --- | --- | --- |
| Mn102 | A | B |
| Mn103 | C | - |
| Mn105 | D | E |

**Table S2:** Polymorphisms that allow the discrimination of the 10 different *Termitomyces* heterokaryons used in the experiments. SNP numerical code corresponds to the position of the polymorphism using the GenBank DQ437019 as EF1-α reference and AB073531 as ITS reference.

| Heterokaryons | EF1-α | | ITS |
| --- | --- | --- | --- |
|  | SNP84 | SNP113 | SNP648 |
| AB | TT | AG | TT |
| AC | CT | AA | TT |
| AD | TT | AA | CT |
| AE | CT | AG | CT |
| BC | CT | AG | TT |
| BD | TT | AG | CT |
| BE | CT | GG | CT |
| CD | CT | AA | CT |
| CE | CC | AG | CT |
| DE | CT | AG | CC |

*On asexual spores and nuclei:*

1. number of nuclei per asexual spore

Asexual spores were scrapped off and immediately placed in 10 μl of the florescent dye DAPI, and we used fluorescence microscopy to visualise the nuclei. A 4,6-diamidino-2-phenylindole dihydrochloride (DAPI) stock solution consisted of 2 mg DAPI in 1 ml DMSO. We have worked with a solution made of 2 μl stock solution mixed with 1 ml 0.1 M Na-phosphate buffer (pH 7.0), for a final concentration of 2 μg·ml^-1^. DAPI binds selectively to AT-rich regions in double stranded DNA and nuclei thus become fluorescent and are visible using fluorescence microscopy (Meixner & Bresinsky 1988, Butt et al. 1989).

1. nuclear genotype of the asexual spores

Our null hypothesis was that nuclei segregate randomly among the asexual spores and the alternative hypothesis that nuclei are distributed in a regulated fashion to maintain two different nuclei per spore. The number of single-spore cultures that need to be genotyped in order to distinguish between these hypotheses was calculated as follows. If nuclei segregate randomly, the probability of a homokaryotic asexual spore depends on the average number of nuclei per spore and on the frequency of the two nuclei in the mycelium. Assuming a binomial distribution, with p=0.5 (which is a conservative estimate), and three nuclei per spore, the probability to obtain a homokaryotic spore is 2*(½)^3=0.25.

**Results**

**Table S3**: Statistic values for the univariate analysis of mycelial growth, GLM ANOVA with karyotic state as main effect, nuclear haplotype as cofactor

| **Between subject factors** | | |
| --- | --- | --- |
|  | | N |
| Karyotic state | hetero | 47 |
|  | homo | 22 |

| **Test of between-subjects effects** | | | | | |
| --- | --- | --- | --- | --- | --- |
| Dependent variable: mycelia linear growth | | | | | |
| Source | Type III  Sum of squares | df | Mean  square | Z | Sig. |
| Corrected model | 752.040^a^ | 6 | 125.340 | 5.573 | .000 |
| Intercept | 3953.880 | 1 | 3953.880 | 175.794 | .000 |
| nA | 147.086 | 1 | 147.086 | 6.540 | .013 |
| nB | 161.463 | 1 | 161.463 | 7.179 | .009 |
| nC | 192.245 | 1 | 192.245 | 8.547 | .005 |
| nD | 137.665 | 1 | 137.665 | 6.121 | .016 |
| nE | 33.951 | 1 | 33.951 | 1.510 | .224 |
| Karyotic state | 321.302 | 1 | 321.302 | 14.285 | .000 |
| Error | 1394.474 | 62 | 22.492 |  |  |
| Total | 121324.801 | 69 |  |  |  |
| Corrected Total | 2146.513 | 68 |  |  |  |
| a. R squared = .350 (adjusted R square = .287) | | | | | |

| **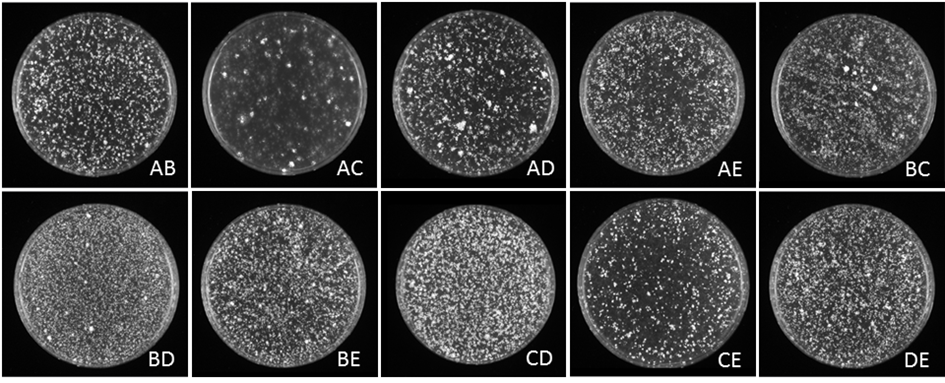** |
| --- |
|  |

**Figure S3:** Representative images of nodule formation in the 10 heterokaryons.

**Table S4**: CFU’s present in the stock suspensions as determined on counting plates and the effective proportions of the different homokaryons in the different combinations.

| homokaryons | cfu / ml |  | codes | targeted combinations | real proportions | | | | | |
| --- | --- | --- | --- | --- | --- | --- | --- | --- | --- | --- |
| A | 1.2E+05 |  | MixA | **4A**:B:C:D:E |  | **4:** | 0.3: | 1.0: | 0.9: | 1.1 |
| B | 3.2E+04 |  | MixB | A:**4B**:C:D:E |  | 1.0: | **1:** | 1.0: | 0.9: | 1.1 |
| C | 1.2E+05 |  | MixC | A:B:**4C**:D:E |  | 1: | 0.3: | **4:** | 0.9: | 1.1 |
| D | 1.1E+05 |  | MixD | A:B:C:**4D**:E |  | 1.1: | 0.3: | 1.1: | **4:** | 1.3 |
| E | 1.4E+05 |  | MixE | A:B:C:D:**4E** |  | 0.9: | 0.3: | 0.9: | 0.8: | **4** |
|  |  |  | MixF | A:B:C:D:E |  | 1: | 0.3: | 1.0: | 0.9: | 1.1 |


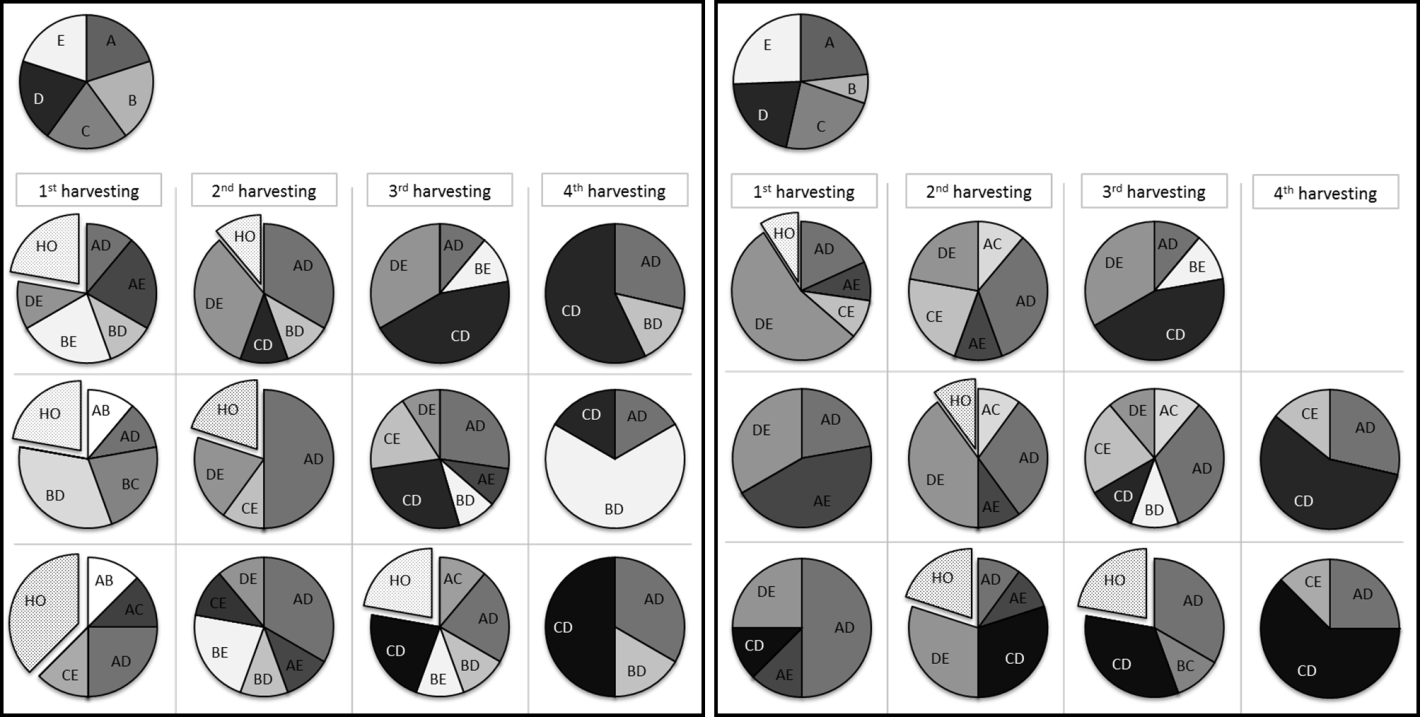


**a)** mix B

**b)** mix F

**Figure S4**: Representation of the different heterokaryons present at each harvesting. Per replicate, a maximum of 10 nodules was sampled (7 ≤ n ≤ 10). At 2^nd^ , 3^rd^ and 4^th^ harvesting, each chart is the representation of the total obtained in 3 plates (3 to 4 nodules sampled per plate). Due to contamination, no data was obtained for one of the replicates of the MixF 4^th^ harvesting. HO: homokaryons.

**
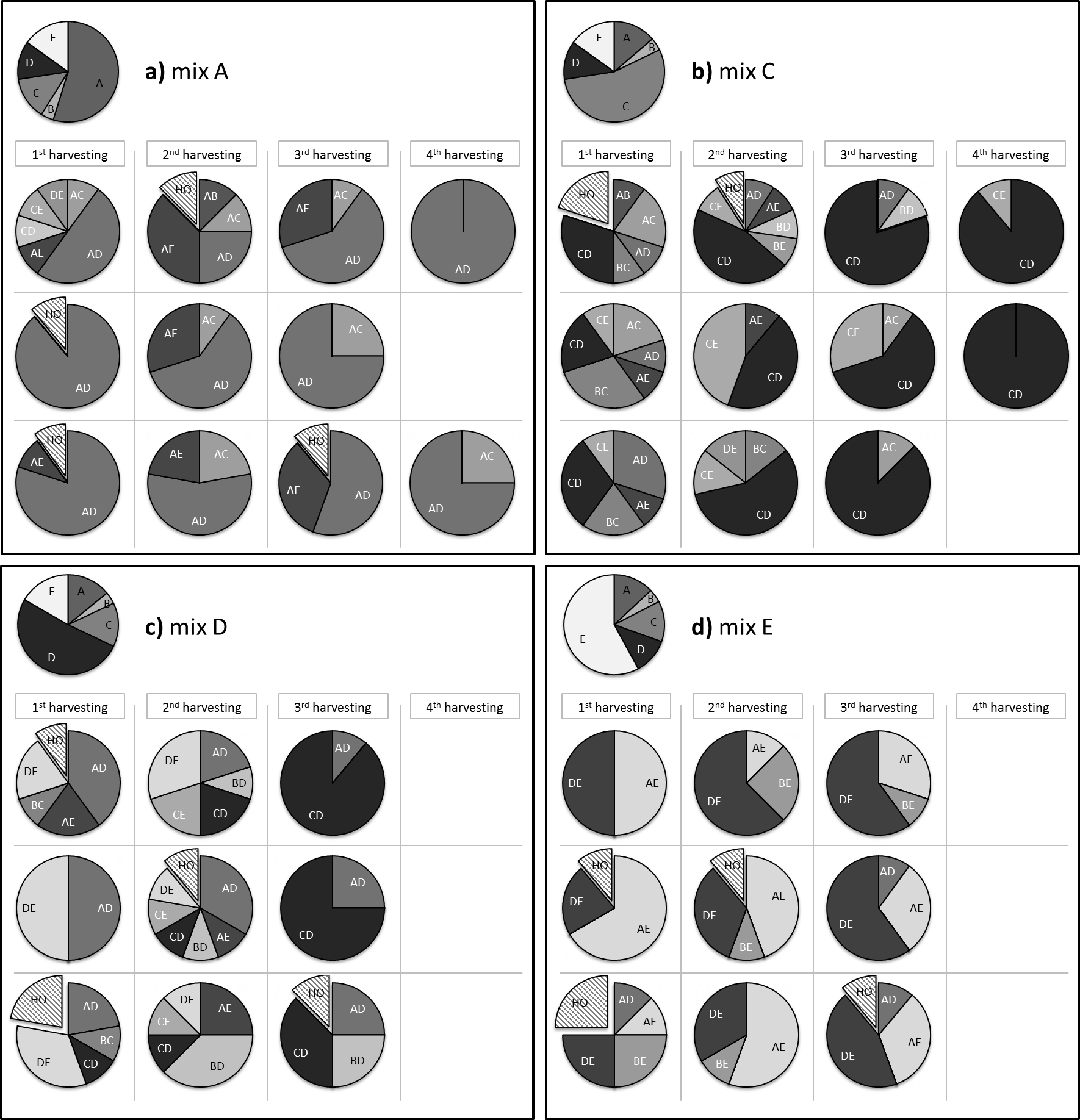
**

**Figure S5**: Representation of the different heterokaryons present at each harvesting. Per replicate, a maximum of 10 nodules was sampled (7 ≤ n ≤ 10). At 2^nd^ , 3^rd^ and 4^th^ harvesting, each chart is the representation of the total obtained in 3 plates (3 to 4 nodules sampled per plate). Due to contamination, reduced amount of data was obtained for the 4^th^ harvesting. HO: homokaryons.

**Table S5:** Simpson diversity index (1-D) per mixture, discriminated following harvesting and replicate. Differences between replicates are significant at p<0.05, following a Student’s t-test.

|  | MixA | Simpson | replicate pair | t | v | p (0.05; v) | sig. |
| --- | --- | --- | --- | --- | --- | --- | --- |
| 1st  harvesting | rep1 | 0.78 | 1-2 | 2.69 | 17.42 | 1.74 | yes |
|  | rep2 | 0.22 | 1-3 | 1.84 | 18.10 | 1.73 | yes |
|  | rep3 | 0.38 | 2-3 | 0.65 | 18.98 | 1.73 |  |
| 2nd harvesting | rep1 | 0.86 | 1-2 | 1.87 | 16.80 | 1.75 | yes |
|  | rep2 | 0.60 | 1-3 | 1.42 | 15.66 | 1.75 |  |
|  | rep3 | 0.67 | 2-3 | 0.42 | 18.99 | 1.73 |  |
| 3rd  harvesting | rep1 | 0.25 | 1-2 | 0.77 | 15.71 | 1.75 |  |
|  | rep2 | 0.43 | 1-3 | 1.92 | 12.96 | 1.78 | yes |
|  | rep3 | 0.64 | 2-3 | 1.15 | 14.15 | 1.76 |  |
| 4th  harvesting | rep1 | 0.00 |  |  |  |  |  |
|  | rep2 |  |  |  |  |  |  |
|  | rep3 | 0.43 |  |  |  |  |  |
|  | MixC | Simpson | replicate pair | t | v | p (0.05; v) | sig |
| 1st  harvesting | rep1 | 0.91 | 1-2 | 0.30 | 19.73 | 1.73 |  |
|  | rep2 | 0.89 | 1-3 | 0.89 | 19.85 | 1.73 |  |
|  | rep3 | 0.84 | 2-3 | 0.63 | 19.98 | 1.73 |  |
| 2nd harvesting | rep1 | 0.78 | 1-2 | 0.30 | 16.37 | 1.75 |  |
|  | rep2 | 0.73 | 1-3 | 0.31 | 14.50 | 1.76 |  |
|  | rep3 | 0.71 | 2-3 | 0.11 | 10.13 | 1.81 |  |
| 3rd  harvesting | rep1 | 0.38 | 1-2 | 1.06 | 17.06 | 1.74 |  |
|  | rep2 | 0.60 | 1-3 | 0.51 | 17.81 | 1.74 |  |
|  | rep3 | 0.25 | 2-3 | 1.67 | 14.15 | 1.76 |  |
| 4th  harvesting | rep1 | 0.22 |  |  |  |  |  |
|  | rep2 |  |  |  |  |  |  |
|  | rep3 | 0.00 |  |  |  |  |  |
|  | MixD | Simpson (1-D) | replicate comparison | t | v | p (0.05; v) | sig. (p<0.05) |
| 1st  harvesting | rep1 | 0.82 | 1-2 | 3.30 | 10.00 | 1.81 | Yes |
|  | rep2 | 0.57 | 1-3 | 0.67 | 18.80 | 1.73 |  |
|  | rep3 | 0.89 | 2-3 | 4.90 | 9.00 | 1.83 | Yes |
| 2nd harvesting | rep1 | 0.87 | 1-2 | 0.63 | 14.00 | 1.76 |  |
|  | rep2 | 0.92 | 1-3 | 0.11 | 11.84 | 1.80 |  |
|  | rep3 | 0.86 | 2-3 | 0.57 | 16.62 | 1.75 |  |
| 3rd  harvesting | rep1 | 0.38 | 1-2 | 0.22 | 17.98 | 1.74 |  |
|  | rep2 | 0.43 | 1-3 | 2.38 | 12.12 | 1.78 | Yes |
|  | rep3 | 0.82 | 2-3 | 2.40 | 10.28 | 1.81 | Yes |
|  | MixE | Simpson | replicate pair | t | v | p (0.05; v) | sig. |
| 1st  harvesting | rep1 | 0.56 | 1-2 | 0.00 | 9.00 | 1.83 |  |
|  | rep2 | 0.56 | 1-3 | 8.81 | 8.00 | 1.86 | yes |
|  | rep3 | 0.89 | 2-3 | 2.14 | 10.12 | 1.81 | yes |
| 2nd harvesting | rep1 | 0.61 | 1-2 | 0.85 | 13.13 | 1.77 |  |
|  | rep2 | 0.75 | 1-3 | 0.18 | 14.64 | 1.76 |  |
|  | rep3 | 0.64 | 2-3 | 0.84 | 17.50 | 1.74 |  |
| 3rd  harvesting | rep1 | 0.60 | 1-2 | 0.00 | 20.00 | 1.72 |  |
|  | rep2 | 0.60 | 1-3 | 1.05 | 18.08 | 1.73 |  |
|  | rep3 | 0.75 | 2-3 | 1.05 | 18.08 | 1.73 |  |
|  | MixB | Simpson | replicate pair | t | v | p (0.05; v) | sig |
| 1st  harvesting | rep1 | 0.94 | 1-2 | 0.75 | 14.26 | 1.76 |  |
|  | rep2 | 0.89 | 1-3 | 0.28 | 16.07 | 1.75 |  |
|  | rep3 | 0.93 | 2-3 | 0.51 | 15.54 | 1.75 |  |
| 2nd harvesting | rep1 | 0.83 | 1-2 | 0.57 | 16.20 | 1.75 |  |
|  | rep2 | 0.76 | 1-3 | 0.58 | 17.90 | 1.74 |  |
|  | rep3 | 0.89 | 2-3 | 1.00 | 15.54 | 1.75 |  |
| 3rd  harvesting | rep1 | 0.75 | 1-2 | 1.26 | 14.25 | 1.76 |  |
|  | rep2 | 0.87 | 1-3 | 1.80 | 11.92 | 1.80 | yes |
|  | rep3 | 0.92 | 2-3 | 0.75 | 19.27 | 1.73 |  |
| 4th  harvesting | rep1 | 0.67 |  |  |  |  |  |
|  | rep2 | 0.60 |  |  |  |  |  |
|  | rep3 |  |  |  |  |  |  |
|  | MixF | Simpson | replicate comparison | t | v | p (0.05; v) | sig. |
| 1st  harvesting | rep1 | 0.71 | 1-2 | 0.09 | 15.29 | 1.75 |  |
|  | rep2 | 0.72 | 1-3 | 0.24 | 18.93 | 1.73 |  |
|  | rep3 | 0.75 | 2-3 | 0.22 | 11.94 | 1.80 |  |
| 2nd harvesting | rep1 | 0.86 | 1-2 | 0.64 | 17.42 | 1.74 |  |
|  | rep2 | 0.80 | 1-3 | 0.07 | 18.90 | 1.73 |  |
|  | rep3 | 0.87 | 2-3 | 0.66 | 18.93 | 1.73 |  |
| 3rd  harvesting | rep1 | 0.80 | 1-2 | 0.87 | 18.62 | 1.73 |  |
|  | rep2 | 0.89 | 1-3 | 0.06 | 16.78 | 1.75 |  |
|  | rep3 | 0.81 | 2-3 | 1.01 | 17.02 | 1.74 |  |
